# Evaluation of Mechanical and Tribological Aspect of Self-Lubricating Cu-6Gr Composites Reinforced with SiC–WC Hybrid Particles

**DOI:** 10.3390/nano12132154

**Published:** 2022-06-23

**Authors:** Üsame Ali Usca, Serhat Şap, Mahir Uzun, Khaled Giasin, Danil Yurievich Pimenov

**Affiliations:** 1 Department of Mechanical Engineering, Bingöl University, Bingöl 12000, Turkey; 2 Department of Electricty and Energy, Bingöl University, Bingöl 12000, Turkey; 3 Department of Mechanical Engineering, İnönü University, Malatya 44000, Turkey; mahir.uzun@inonu.edu.tr; 4 School of Mechanical and Design Engineering, University of Portsmouth, Portsmouth PO1 3DJ, UK; 5 Department of Automated Mechanical Engineering, South Ural State University, Lenin Prosp. 76, 454080 Chelyabinsk, Russia; danil_u@rambler.ru

**Keywords:** copper matrix composites, graphite, hardness, specific wear rate, bending stress

## Abstract

Because of their high thermal conductivity, good corrosion resistance, and great mechanical qualities, copper matrix composites are appealing materials utilized in a variety of industries. This study investigates the mechanical properties of copper–graphite (Cu–Gr) matrix composites reinforced with silicon carbide (SiC) and tungsten carbide (WC) particles by hot pressing using powder metallurgy method. The goal is to investigate the influence of the reinforcement ratio on the mechanical characteristics of copper composite materials generated (density, hardness, flexural strength, and wear resistance). SEM, EDS, and X-RD analysis were used to perform metallographic examinations. The highest relative density with a value of 98.558% was determined in the C3 sample. The findings revealed that when the reinforcement ratio was raised, the hardness rose. The highest hardness value was observed in the C6 sample with an increase of 12.52%. Sample C4 (with the lowest SiC and WC particles ratio) had the highest bending stress (233.18 MPa). Bending stress increased by 35.56% compared to the C1 sample. The lowest specific wear rates were found in the C4 sample, with a decrease of 82.57% compared to the C1 sample. The lowest wear rate (6.853 × 10^−7^ mm^3^/Nm) also occurred in the C4 sample. The microstructural analysis showed that the hybrid reinforcement particles exhibited a homogeneous distribution in the copper matrix. X-RD analysis showed that there was no intermediate reaction between the parent matrix and the hybrid reinforcements. A good interfacial bond was observed between the matrix structure and the hybrid reinforcements. The motivation of this research was to utilise the advantages of the unique features of SiC–WC hybrid particles to improve the performance of newly developed Cu-6Gr composites for wear-resistance applications.

## 1. Introduction

Pure copper is known to have weak mechanical properties (hardness, strength, wear resistance), which limits its applications in the field of engineering [1]. Despite this, it is seen that copper is widely used in many areas of industry. From the past to the present, copper appears in the form of many different products. Some of those: brass, bronze, copper–nickel, and copper–titanium alloys can be given as examples. Therefore, copper is usually reinforced with other materials in the form of particles to form copper matrix composites [2]. These composites provide improved material properties over monolithic copper material. By altering the type of reinforcements and their ratios, the mechanical, thermal, and electrical properties of the copper composites can be customed to suit different applications and industries. Reinforcement particles with a stable structure can thermodynamically improve the mechanical performance of copper matrix composites but have less effect on electrical properties [3]. In recent years, copper matrix composite materials were preferred in many fields, such as engineering materials, thanks to their superior mechanical properties [4]. Copper composites, which are formed by the combination of the excellent elastic modulus of ceramic materials and the high ductility of copper, are used especially in the aerospace and automotive industries [5]. Copper matrix composites are widely used in many fields, such as electrical household appliances, aerospace, automotive and electronics industries [6]. In addition, Cu–Gr/SiC composites are used in bearings, bushings, and heat sinks due to their low thermal expansion coefficient [7]. Copper matrix composite possess excellent properties, including good thermal-electrical conductivity, high mechanical strength, and corrosion [8]. As reinforcement materials, many powder particles, such as SiC (silicon carbide) [9], Al_2_O_3_ (aluminium oxide) [10], WC (tungsten carbide) [11], B_4_C (boron carbide) [12], Ti (titanium) [13], TiC (titanium carbide) [14], and TiB_2_ (titanium diboride) [3] are used. Akbarpour and Alipour [15], fabricated copper composites reinforced with SiC nanoparticles and studied their wear and friction behaviour under dry sliding conditions. As a result of the experiments, they reported that the wear scar and friction coefficients were reduced by adding 4 vol.% SiC to the copper matrix. Metal matrix composite materials show stable tribological properties over prolonged periods of time even in different environments and high temperatures [16]. Pradhan and Das [17], fabricated Cu–SiC composites and examined their wear and friction performance under dry sliding conditions. They reported that the wear rate of the produced nano composites decreased with the increase in shear rate. There are many studies investigating the mechanical and tribological performances of copper matrix composites [18]. Uzun et al. [19] reported that the corrosion performance of the copper composites they produced by adding CrC to pure copper increased. Nageswaran et al. [20] investigated the mechanical and wear behaviour of Cu-TiO_2_-Gr composites. As a result, it was reported that the mechanical and wear properties of the produced composite were improved. However, these studies are insufficient. Graphite powders are added to the copper matrix along with reinforcements to increase the wear performance [21]. The purpose is to form a lubricating film layer between the graphite disc and the material during wear and to reduce the wear rate. Nayak et al. [22] reported that the wear rates decreased with the increase in graphite and titanium carbide reinforcements in the copper matrix. They also reported that the increase in TiC reinforcement caused an increase in the hardness of the hybrid composites. Li et al. [23], investigated the mechanical and wear behavior of Cu-GNS and Cu-graphite by hot pressing method. As a result of the addition of graphene nanoplates, they achieved higher mechanical properties and wear performance. Having a laminar structure of graphite affects the tribological properties of metal matrix composites and helps to reduce the friction coefficient with its lubricating property [24]. At the same time, graphite powder particles increases the thermal and electrical performance of composites [25]. WC, which was used as the second reinforcement in the current study, was preferred because of its high hardness and resistance to high temperatures. In addition, the wettability of copper with WC reinforcement particles was proven to be better than many carbides [26].

In previous studies, copper matrix composites with lubricating graphite and a single reinforcement were produced. However, there were no studies on the addition of hybrid reinforcement for Cu–Gr composites. It is known in the literature that SiC-WC reinforcements improve mechanical properties in composite materials. It was aimed to increase the mechanical and tribological performances of Cu–Gr composites with added reinforcement particles (SiC-WC). In this study, the combined effects of reinforcements were investigated by adding additional reinforcing element WC to Cu–Gr/SiC composites. Metallographic analysis (SEM, EDS, X-RD) of the composites produced by hot pressing were performed. Density, hardness, three-point bending, and wear analysis were evaluated to determine the effect of the reinforcement ratio.

## 2. Materials and Methods

**Figure 1:**
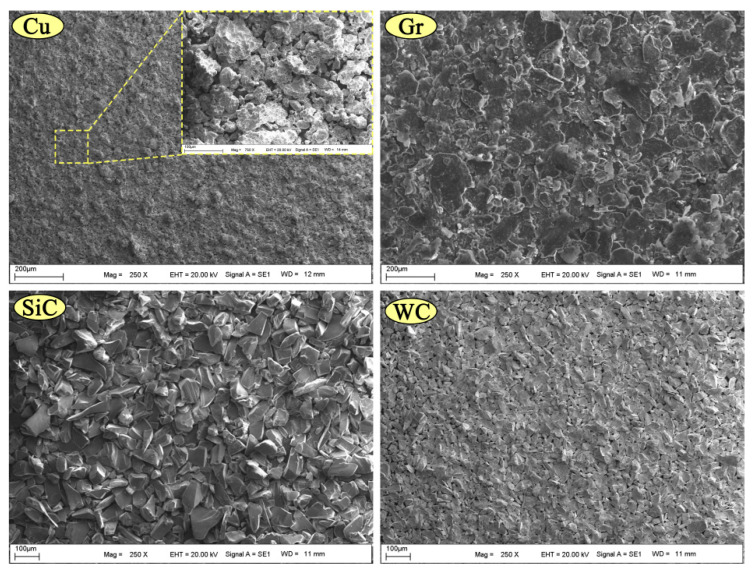
Micrographs of the powders used in the tests taken using a SEM.

Pure copper with an average particle size of 45–75 µm was used as a matrix material in the production of composites. To increase the wear performance, Gr powder with an average particle size of 44 µm was added to the matrix at a fixed rate (6 wt.%); the powder is known to have lubricating properties. Figure 1 shows SEM graphs of the dust particles used in the experiments. It is known that graphite powders added to copper reduce mechanical properties [27]. To improve the mechanical properties, Cu-6Gr/SiC-WC compacts were prepared by adding SiC and WC reinforcement powders at different rates into Cu-6Gr powders. Some properties of the powder particles used are shown in Table 1. In Table 2, the mixing ratios of the selected powder particles are given.

Ege Nanotek (Istanbul, Turkey) provided metal powders of high purity. To remove any moisture, the powders were dried in a drying oven at 100 °C. Weighing of the powder particles was carried out using a precision balance (0.0001 gr). By adding 1.5 wt.% Peg400 (polyethylene glycol) into the mixture, the powders were prevented from falling out of the graphite mould. The powders were mixed for approximately 25 min using a Turbula (Cel-mak Group 7T, Elazig, Turkey) to obtain a good dispersion. In order to prevent agglomeration, 1/3 steel balls were used in the mixing process. The obtained mixture powders were poured into graphite moulds and sintered in a PLC-controlled hot press machine (Zhengzhou Golden Highway, SMVB80, Zhengzhou, China) at 850 °C, under 35 MPa pressure, for 6 min to produce parallelepiped composite samples (40 × 15 × 10 mm^3^). Protective nitrogen gas was used in a controlled rate to mitigate oxidation that may occur as a result of high temperature. The densities of the obtained composites were determined by the Archimedes principle. Density values were calculated using Equations (1)–(3).

(1) $$\rho_{\mathrm{theoretical}\;}{=(w}_{\mathrm{Cu}}{\cdot \rho }_{\mathrm{Cu}}{)+(w}_{\mathrm{Gr}}{\cdot \rho }_{\mathrm{Gr}}{)+(w}_{\mathrm{SiC}}{\cdot \rho }_{\mathrm{SiC}}{)+(w}_{\mathrm{WC}}{\cdot \rho }_{\mathrm{WC}})$$

(2) $$\rho_{\mathrm{experimental}\;}=\left(\frac{m_{\mathrm{air}}}{m_{\mathrm{air}}-m_{\mathrm{water}}}\right){\cdot \rho }_{\mathrm{water}}$$

(3) $$\rho_{\mathrm{relative}\;}=\left(\frac{\rho_{\mathrm{experimental}\;}}{\rho_{\mathrm{theoretical}\;}}\right)\cdot 100$$

Here, W: weight fraction, ρ: material density, $\rho_{\mathrm{water}}$: density of distilled water (0.998 g/cm^3^ at 20 °C), $m_{\mathrm{air}}$: weight of sample in air,$\;m_{\mathrm{water}}$: it represents the weight of the sample in water. Metallographic analyses of composite samples were carried out in a JEOL JSM 6510 SEM. The samples were first sanded. The samples, whose surfaces were made flat and smooth, were polished with a 3-micron diamond solution. To determine the grain boundaries in composite structures and to obtain quality SEM photographs, the polished surfaces were cauterized using the solution we prepared (25 mL HCl 1–2 gm Fe_3_-Cl-100 mL H_2_O) [28]. After the cauterization process, metallographic analysis was performed on the composite samples and the results were evaluated. After microstructural analysis, hardness analysis was applied to the samples. Hardness analysis on the EMCO TEST Durajet device (EMCO company, Kuchl, Austria) was performed using the Brinell method. Contacting both the matrix and the reinforcements with the ball employed in the Brinell macro hardness analysis method yields more efficient findings. Initially, a 2.5 mm diameter ball was used to apply a 10 kg preload to the samples with flat and smooth surfaces. Then, by applying a load of 31.25 kg, the macro hardness results were determined and recorded. Each hardness measurement was repeated 5 times to minimize the margin of error in the experiments. A three-point bending test was applied to measure the strength of the produced composites. An electronic universal testing machine (SHIMADZU AGS-X, Duisburg, Germany) with a tensile and compression capacity of 50 kN was used for this experiment. The tests were carried out at room temperature at a compression speed of 1 mm/min, and the experiments were repeated three times for each different ratio in order to reduce the margin of error. The support distance of the samples prepared for the three-point bending test was adjusted to 30 mm according to ASTM D 790 standards. The specimens, whose midpoints were determined, were placed between two supports and the compression process was started. The compression process in the device was continued automatically until the sample was broken. The results were recorded, and the maximum bending strength was calculated according to Equation (4). After the three-point bending test, SEM analyses were performed to determine the structure and crack morphology on the fractured surfaces of the samples.

(4) $$\sigma =3\cdot F\cdot L/2b\cdot d^{2}$$

**Figure 2:**
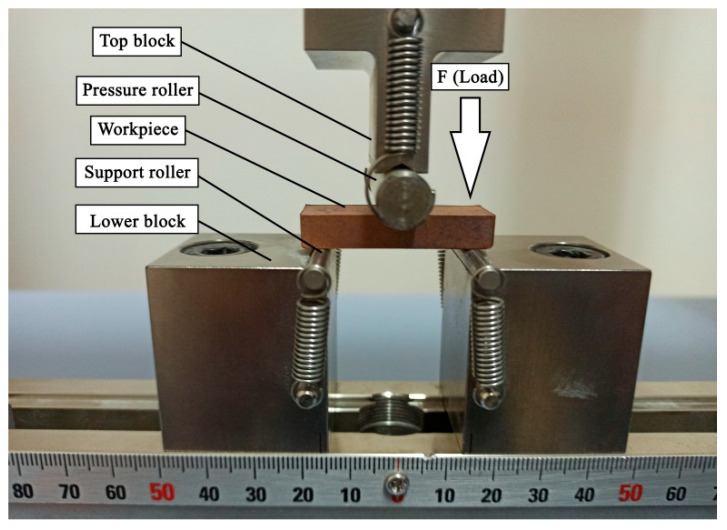
Image of C1 specimen during three-point bending test.

In this equation, σ: bending stress (MPa), F: applied load (N), L: the distance between the two supports (mm), b: width of the sample (mm), and d: thickness of the sample (mm). Figure 2 shows the photograph of sample C1 during the three-point bending test.

Abrasion tests were performed to determine the tribological properties of the produced composites. The tests were carried out at room temperature and under dry sliding conditions. Abrasion tests were performed on a pin-on-disc tribological test device (TURKYUS, Bursa, Turkey) in accordance with ASTM G99-95 standards. The disc was made by wire erosion and toughened by surface nitriding using AISI D2 tool steel as the counter surface. Before starting the tests, the hardness value of the abrasive discs was determined as 756 HV (62.4 HRC). The average surface roughness of the etching discs was determined as 4.5 µm. Each experiment was repeated 3 times to reduce the margin of error in the experiments. Before each wear test, the disc and specimen surfaces were cleaned with ethyl alcohol. The studies were conducted out under dry sliding circumstances using a 1000 m road, a wear rate of 1.5 m/s, and a weight of 10 N. During the wear test, friction forces were measured using a strain gauge linked to the tribo test equipment, and the average friction coefficient was calculated using Equation (5).

(5) µ=F/N

In this equation, µ: average friction coefficient, F: friction force (N), and N: the amount of load (N) applied during the wear test. The results were saved to the device. Following the wear test, the mass losses of each sample were calculated. The following formulae were used to compute the SWR (Specific Wear Rate) value for each sample (Equations (6) and (7)).

(6) $$V_{L}{=\Delta }_{m}/\rho$$

(7) $${\mathrm{SWR}=V}_{L}/(N\cdot X)$$

**Figure 3:**
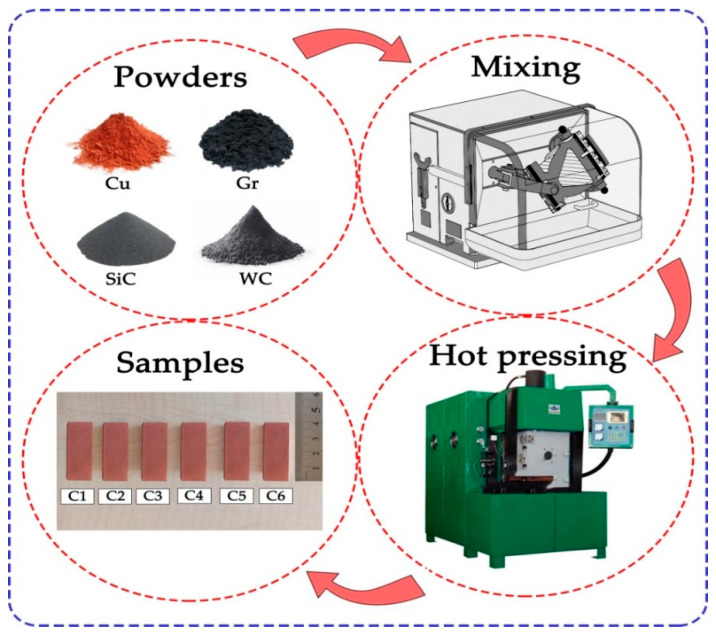
Fabrication scheme of composites.

$V_{L}\;$ is the total volume loss (mm^3^), $\Delta_{m}$ is the mass loss (g), ρ: density (g/cm^3^), N is the load (N) applied during the wear test, X is the wear distance (m), and SWR is the specific wear rate (mm^3^/Nm). Furthermore, the temperature creation between the rubbing objects during the wear test was measured. At 200 m intervals, 5 separate temperature measurements were collected from each sample using a thermal camera (Testo SE & Co. KGaA, Bangkok, Tayland). Figure 3 depicts a test chart of the composite sample fabrication processes.

## 3. Results

### 3.1. Composite Structures Morphology Evaluation

**Figure 4:**
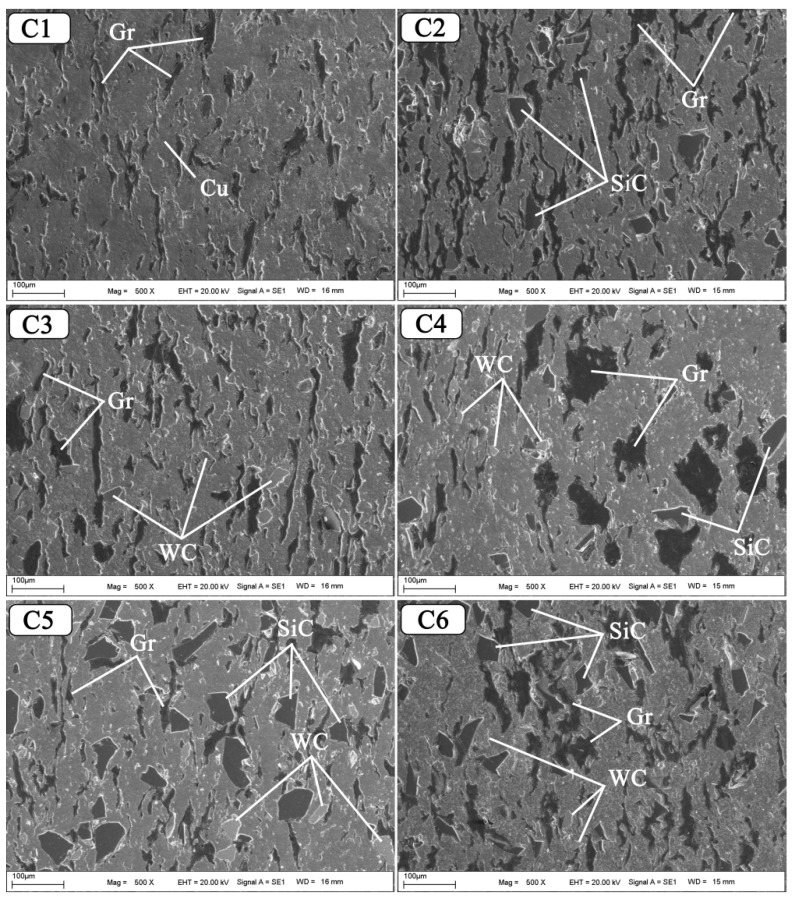
Composite SEM graphs manufactured at various reinforcement ratios.

The SEM photographs of Cu-6Gr/SiC-WC composites produced by hot pressing using the powder metallurgy method are shown in Figure 4. Large amounts of Gr, SiC, and WC particles are visible as dark-coloured regions in composite structures. As shown in the SEM graphs, the SiC particles in the structure led to the uniform distribution of WC in the composite samples [29]. According to the images obtained from the surface photographs of the composites, it can be said that the reinforcements exhibit a good distribution within the structure. No voids or cracks were observed on the surfaces as a result of sintering by hot pressing. In addition, a non-porous and dense structure was observed in the hybrid composites. It is seen that the graphite has a tail-shaped aspect in the structure. SiC and WC particles used as reinforcements are also seen embedded in the matrix structure. In addition, the matrix and hybrid reinforcements were found to have a good interfacial bond. A good interface and homogeneous dispersion are required to increase tribological performance [30].

**Figure 5:**
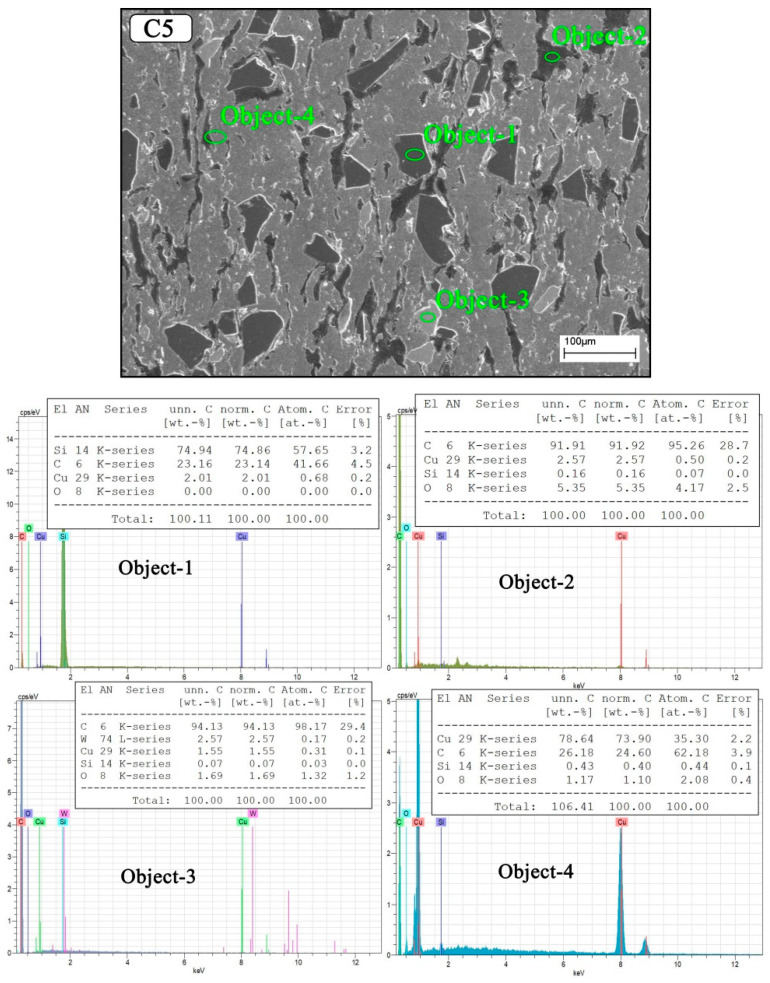
SEM/EDS images of copper composite sample C5.

Figure 5 shows the SEM/EDS analysis taken from the surface of hybrid reinforced (Gr–SiC–WC) copper composite samples. The region indicated by object 1 represents SiC, the region designated as object 2 is Gr, the region indicated by object 3 is WC, and the region indicated by object 4 represents a mixed copper-rich region. Thus, the existence of graphite and hybrid reinforcements in the composite structures is confirmed. In addition, the atomic weights of the particles in the EDS areas were given in the form of a table. Meher and Chaira [31] produced Cu–Gr/SiC composites by powder metallurgy method. As a result of their EDS analysis, they examined the quantitative analysis of three different phases. They reported that the highest peaks were Cu, C, and Si elements.

**Figure 6:**
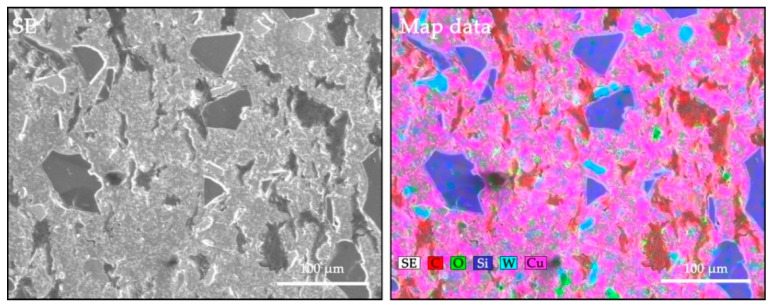
Mapping analysis micrographs of C4 composite sample.

To determine the existence and dispersion of particles within the composite structures, a component mapping analysis was done (Figure 6). The copper matrix is represented by the pink-coloured areas, the graffiti by the red-coloured areas, the silicon carbide by the dark blue coloured areas and the tungsten carbide by the blue coloured areas. Particles whose existence was proven by EDX analysis were coloured by elemental mapping.

**Figure 7:**
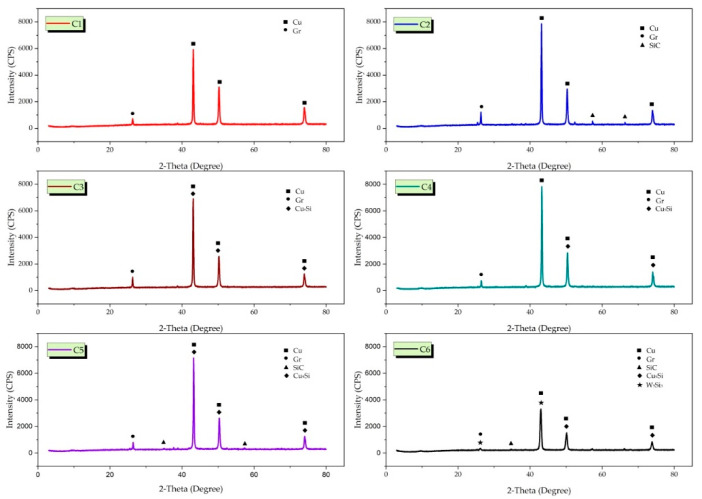
X-RD plots of composite specimens.

Figure 7 and Supplementary File shows the X-RD patterns of Cu-6Gr/SiC-WC composites produced by hot pressing. The characteristic peaks of copper are generally seen in the X-RD model. Due to the low sintering temperature (850 °C), SiO and CuO did not form, and no peaks were found as a result of the analysis. This is one of the superior aspects of hot pressing. In X-RD analysis, Cu, Gr (C), SiC, Cu_9_Si (silicon copper) and W_5_Si_3_ (silicon tungsten) phases were detected in composite structures. It is seen that the peak intensity decreases in some samples with the addition of reinforcement particles. This decrease is attributed to the thermal expansion coefficient between the Cu matrix and the secondary phase SiC particles. A similar study supports this opinion [32]. Powder particles used at the micro-level by hot pressing have little effect on grain growth. At the same time, hybrid supplements significantly reduce grain growth with a homogeneous distribution [33]. Silicon carbide powder particles enter the copper grain boundaries, fixing them, and preventing grain growth [34].

### 3.2. Density Tests

**Figure 8:**
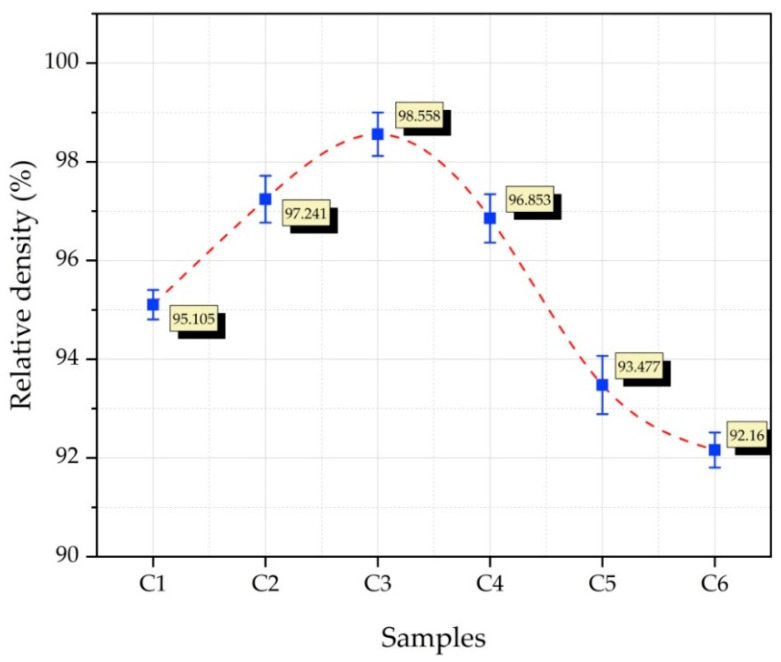
The composite samples’ relative density graph.

The relative density values of Cu composites reinforced with Gr, SiC, and WC powder particles are given in Figure 8. The lowest (92.16%) and highest (98.558%) densities were found in samples C6 (Cu-6Gr/3SiC-3WC) and C3 (Cu-6Gr/4WC), respectively. It is known that the reinforcement elements added into the matrix structure generally reduce the density [6]. The density of composite material can be affected by many parameters, such as the matrix, the structure of the particles, and the production method applied [35]. In our previous study, it was found that the hybrid reinforcements (Ti–B–SiC) added to copper decreased the density after a certain ratio [36], which was also reported in previous studies [37].

### 3.3. Hardness Analysis

**Figure 9:**
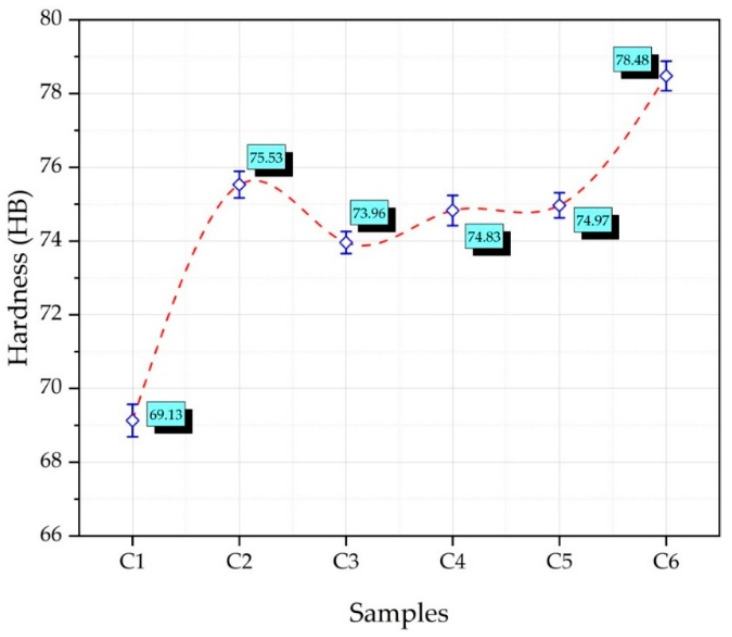
The resulting composite specimens’ macro hardness plot.

Figure 9 shows the Brinell macro hardness of the composite samples produced at different reinforcement ratios by hot pressing. It was observed that there was a general increase in the hardness with the increase in the reinforcement ratio. Moustafa and Taha [38] investigated the mechanical properties of the composites by adding silicon carbide to copper. Their findings revealed that when reinforcing ratios rose, so did hardness. The highest hardness was 78.48 HB in the sample C6 (Cu-6Gr/3SiC-3WC). The lowest hardness 69.13 HB was recorded in the C1 (Cu-6Gr) sample. It is seen that the hardness of the C3 (Cu-6Gr/4WC) sample has decreased compared to the C2 (Cu-6Gr/SiC) sample. From this perspective, SiC particles have a higher impact on hardness. Meher and Chaira [31] investigated the mechanical properties of the composites produced by adding Gr and SiC to pure Cu. They reported that the Gr particles reduced the hardness, while adding SiC increased it. Usca et al. [39], created composite materials by combining B–CrC hybrid reinforcements in various ratios (0-2.5-5-7.5 wt.%) with copper. They discovered that increasing the reinforcement ratios resulted in an increase in hardness.

### 3.4. Flexural Strength Analysis

**Figure 10:**
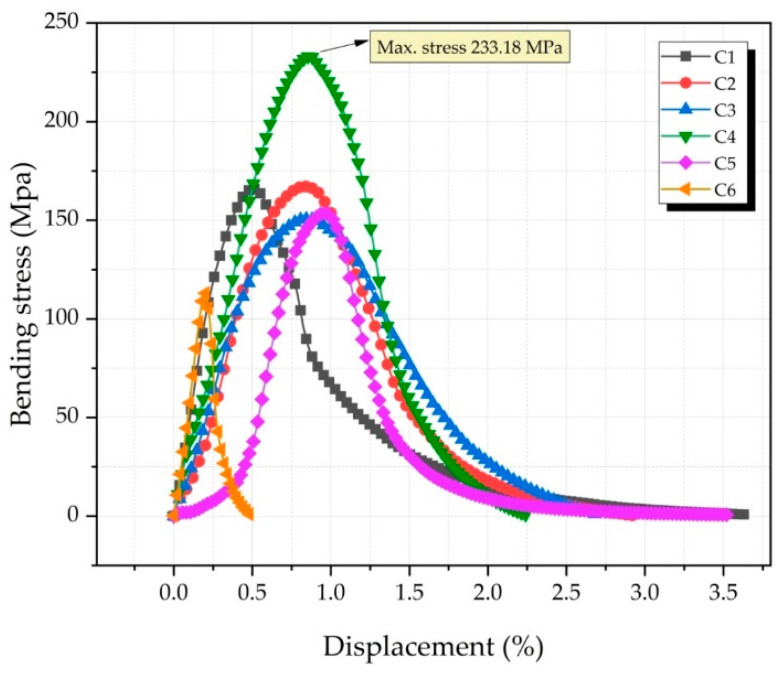
Three-point bending analysis graph of composites.

Three-point bending tests were performed to determine the flexural strength of the produced composites (Figure 10). Sample C4 (Cu-6Gr/1SiC-1WC) had the highest bending stress (233.18 MPa). The lowest bending stress (113.07 MPa) was observed in the C6 (Cu-6Gr/3SiC-3WC) sample. The decrease in the bending stress of the sample C6 can be attributed to the hard and brittle nature of the composite. In composites reinforced with ceramic reinforcements, the shape of the particles, and the matrix interface bonds seriously affect the mechanical properties [40]. A good interfacial bond is needed for the reinforcement elements to enhance the mechanical properties of the composite. The interfacial bond may deteriorate with the increase in the reinforcement content [32]. Due to the thermal incompatibility of the primary matrix and reinforcing components, adverse scenarios may emerge. Şap et al. [36] applied a three-point bending test to the hybrid composite specimens produced by adding Ti-B-SiC at certain rates (0-2-4-6-8 wt.%) into pure copper. Their results showed that the sample containing 4 wt.% hybrid reinforcements had the highest bending strength. Xiao et al. [41] produced WC-reinforced Cu–Sn–Ti hybrid matrix alloys with different ratios (0-15-20-25-30-35 wt.%). They determined that the highest bending strength was in the sample containing 25 wt.% WC.

**Figure 11:**
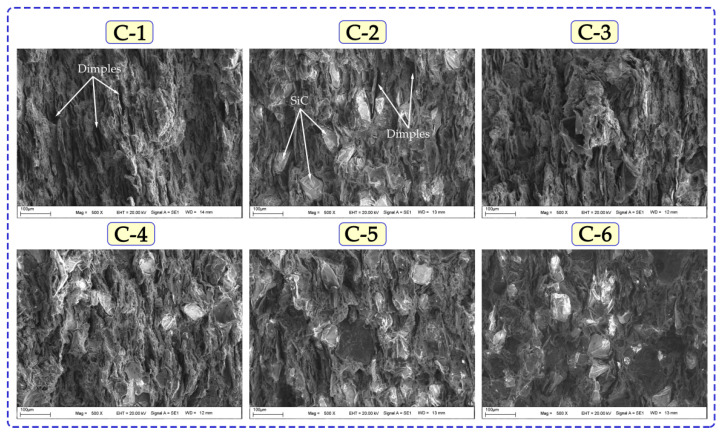
Fractured surface SEM micrographs after the three-point bending test.

Figure 11 shows the fracture surfaces obtained after the three-point bending test of composites containing Cu-6Gr and hybrid reinforcements. The SEM graphs show an increase in reinforcement rates. Height differences were clearly seen in the fracture surface SEM micrographs. In addition, the presence of micro and macro pores was detected. This porosity was shown to grow in lockstep with density. Pits and voids resembling ductile fracture are observed on the surface of the sample C1 (Cu-6Gr). A more brittle fracture is observed on the fracture surfaces of hybrid and mono-reinforced composites. It can be seen that the surfaces of reinforced composites have a flatter structure. Sap et al. [36], in a study they conducted, determined that the brittle fracture mechanism was more pronounced as the reinforcement ratio increased. They also observed that the number of micro and macro pores varied in parallel with the composite density.

### 3.5. Wear Analysis

**Figure 12:**
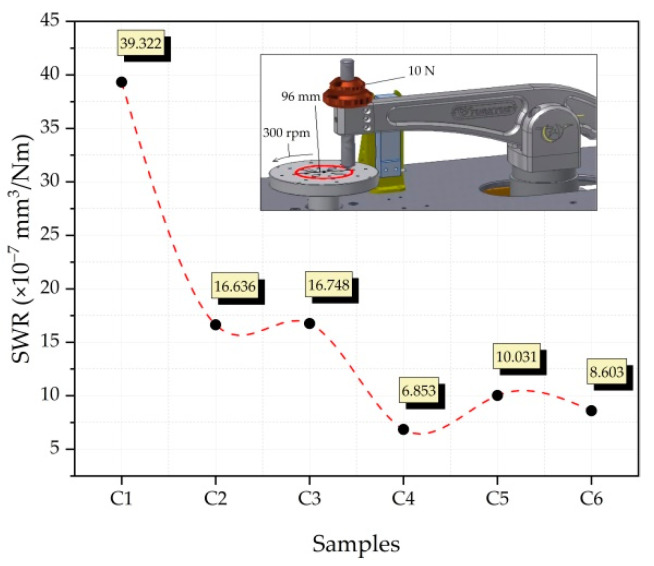
Specific wear rate graph resulting from the wear test.

Figure 12 shows the SWR graph obtained at the end of the wear analysis of the produced composite samples. It gives important information about total volume loss, applied load, density, and wear distance [42]. When the graph is examined in general, the abrasion resistance was increased considerably with the addition of single and hybrid reinforcements. When the C2 (Cu-6Gr/4SiC) and C3 (Cu-6Gr/4WC) samples are examined, it is seen that the C2 sample has better abrasion resistance. The lowest wear rate was recorded as 6.853 × 10^−7^ mm^3^/Nm in the C4 (Cu-6Gr/2SiC-2WC) sample. The highest wear rate was observed in the C1 (Cu-6Gr) sample as 39.322 × 10^−7^ mm^3^/Nm. According to the results obtained, it is seen that the C4 sample has the best wear resistance, which is also evident from the hardness results. Graphite particles can help increase wear resistance by reducing friction and forming a lubricating layer on the opposite surface during wear analysis [43]. It was previously reported that with the homogeneous distribution of SiC particles used as reinforcement, plastic deformation in the composite will be prevented and wear rates will decrease [6]. A good interfacial bond between the reinforcement particles and the matrix can also lead to increased wear resistance [44]. From this point of view, it can be said that the interfacial bond in hybrid composites is good and, therefore, increases the wear resistance.

**Figure 13:**
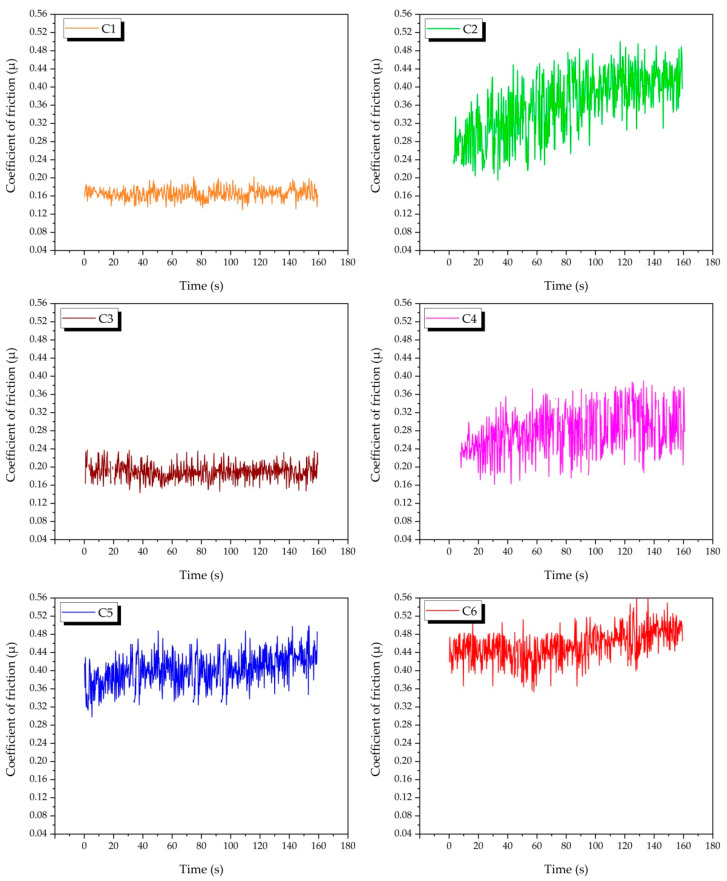
Time-dependent variation of the coefficient of friction of composite samples.

**Figure 14:**
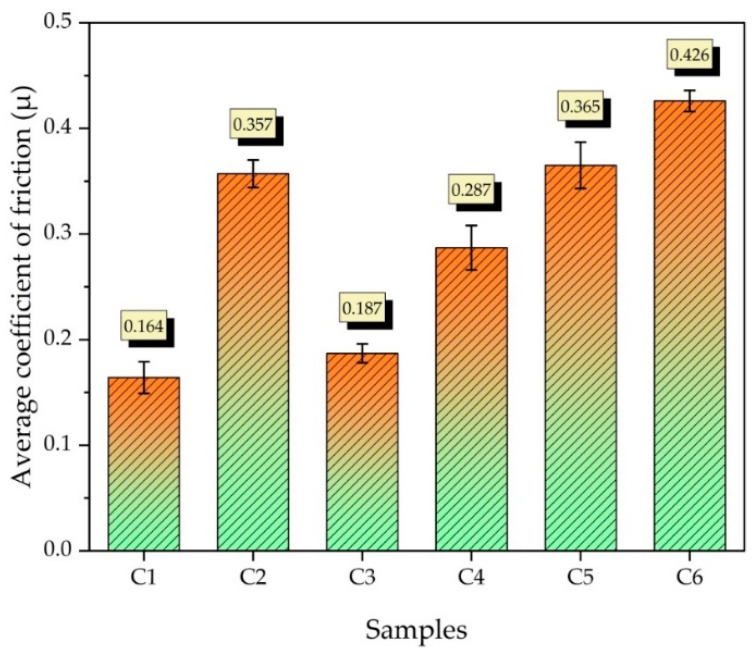
Average friction coefficient of samples produced at different ratios.

Figure 13 shows the graphs of the change in the coefficient of friction of all samples depending on the slip time. The average creep coefficient graph is shown in Figure 14. The lowest average friction coefficient was recorded as 0.164, and the highest average friction coefficient was recorded as 0.426. The low yield strength of copper can increase the wear rate by facilitating crushing during the friction of the two surfaces [45]. When the C2 (Cu-6Gr/4SiC) and C3 (Cu-6Gr/4WC) samples are compared, it is seen that the C3 sample has a lower friction coefficient. When the graphics were analysed in general, it was discovered that the friction coefficients rose as the reinforcement ratio increased. This is due to the fact that the contact between the composite and the counter surface decreased with the increase in the presence of hybrid reinforcements [46].

**Figure 15:**
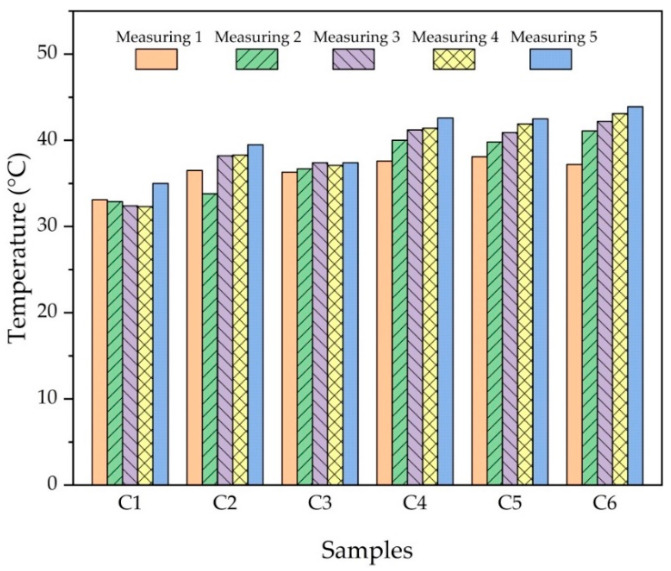
Temperature change on the sample surface during the wear test.

Figure 15 represents the temperature graph generated during the wear tests. Temperatures were recorded by measuring every 200 m of wear distance. When the graph is inspected, it is clear that the rising temperatures are directly proportional to the reinforcement ratios. It was observed that the temperature also increased in each measurement interval. The highest temperature was recorded as 41.4 °C in sample no C6 (Cu-6Gr/3SiC-3WC). The fact that the C6 sample has the highest average friction coefficient supports this situation (highest temperature). As the friction coefficient increases, the temperature also increases.

## 4. Conclusions

In this study, double-reinforced copper composites were produced with the lubricant graphite, which has not been encountered before in the literature. The current study investigates the effect of adding SiC–WC particles to Cu-6Gr composites. The aim is to study the effect of the reinforcement ratio on several mechanical properties of the newly developed Cu-6Gr/SiC-WC composites. Density, hardness, wear rate, and flexural strength were evaluated, and the effect of reinforcement ratio was determined. SEM and EDS analysis were performed to study the developed composites at a microstructural level and explain the trends observed from the analysis of the mechanical properties of the fabricated composites. The following results were found from the study:

- From the SEM graphs, it was observed that the reinforcement elements exhibited a homogeneous distribution in the matrix. In addition, it was determined that the hybrid reinforcements in the structure have a good interface bond with the matrix. Particles in the composite structure were determined by EDS and Mapping analysis. The phases occurring in the structure were determined by X-RD analysis.
- It was observed that the hardness of composite materials increased with the increase in reinforcement particles (SiC–WC), which are harder than copper main matrix and lubricating graphite.
- The highest relative density (98.558%) was detected in the C3 composite, with an increase of approximately 4% compared to the C1 composite.
- As a result of the three-point bending analysis, the highest bending stress was recorded as 233.18 MPa in the C4 sample. It was observed that increasing the reinforcement ratios increased the flexural strength up to a certain level, after which increasing the reinforcement ratio further tended to decrease it.
- Sample C4 exhibited the best wear performance with the lowest SWR value (6.853 × 10^−7^ mm^3^/Nm). The friction coefficient and temperature values increased with increasing reinforcement ratio.

## Figures and Tables

**Table 1:** Some powder particle characteristics.

Powders	Density (g/cm^3^)	Temperature of Melting (°C)	Size of the Particles (µm)	Purity (%)
Cu	8.96	1083	45–75	≥99.90
Gr	2.26	3652	<44	≥98.00
SiC	3.21	2730	45–70	≥99.00
WC	15.63	2870	<44	≥99.20

**Table 2:** The reinforcement ratios of composite samples.

Samples No	Cu (wt.%)	Gr (wt.%)	SiC (wt.%)	WC (wt.%)
C1	94	6	-	-
C2	90	6	4	-
C3	90	6	-	4
C4	92	6	1	1
C5	90	6	2	2
C6	88	6	3	3

## Data Availability

Not applicable.

## Supplementary Materials

The following supporting information can be downloaded at: https://www.mdpi.com/article/10.3390/nano12132154/s1, JCPDS-PDF files for all samples were included as Supplementary file.
