# Supplementary figures and images for: Evaluation of Mechanical and Tribological Aspect of Self-Lubricating Cu-6Gr Composites Reinforced with SiC–WC Hybrid Particles
Source: Nanomaterials (Basel). 2022 Jun 23;12(13):2154. doi: 10.3390/nano12132154 (PMC9268748; doi:10.3390/nano12132154)

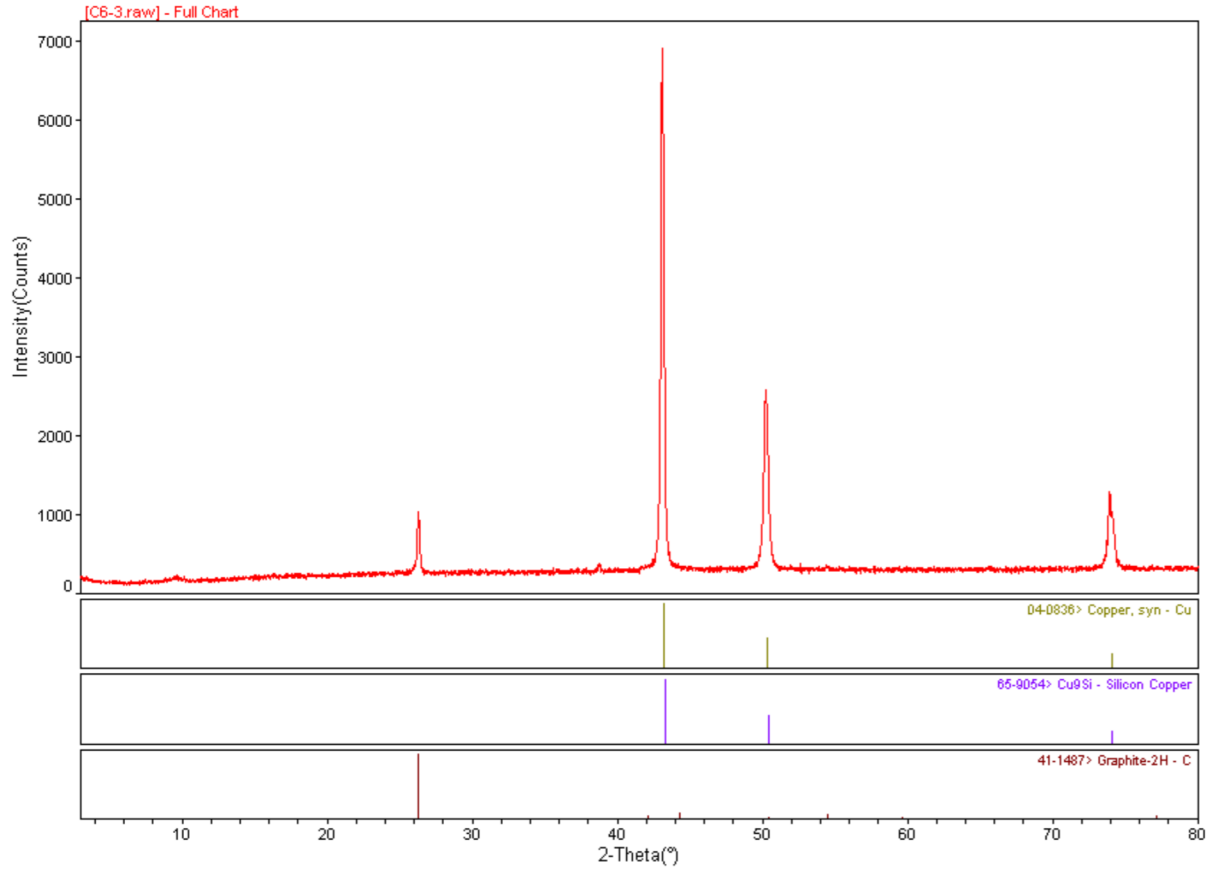

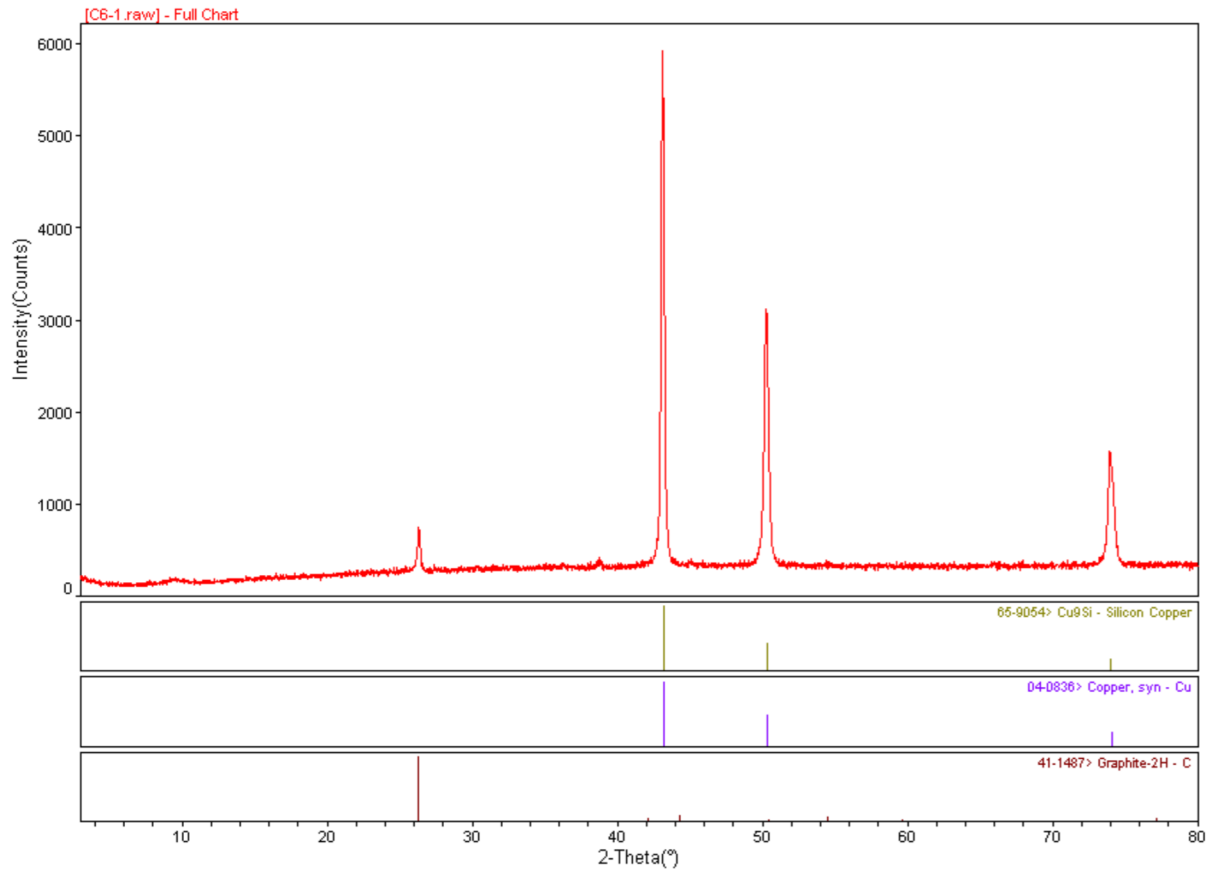

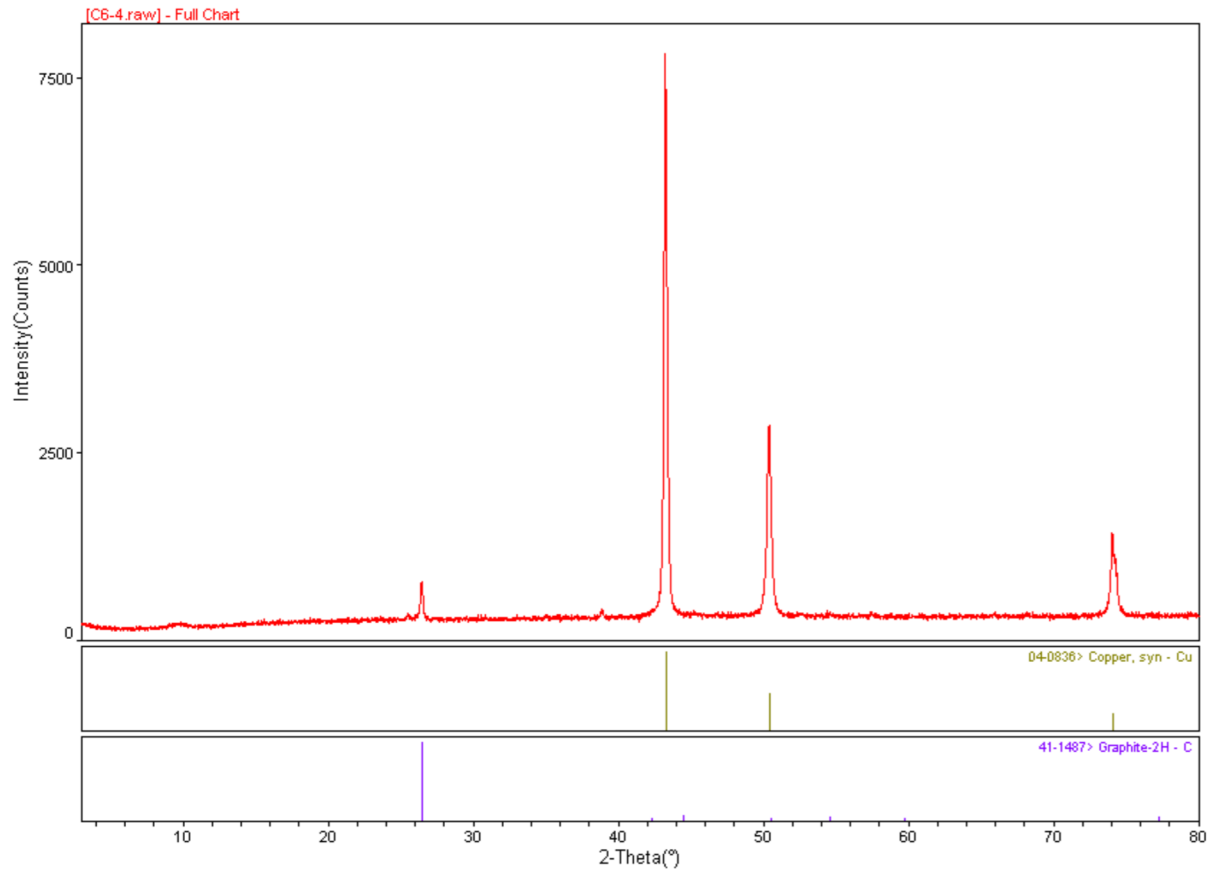

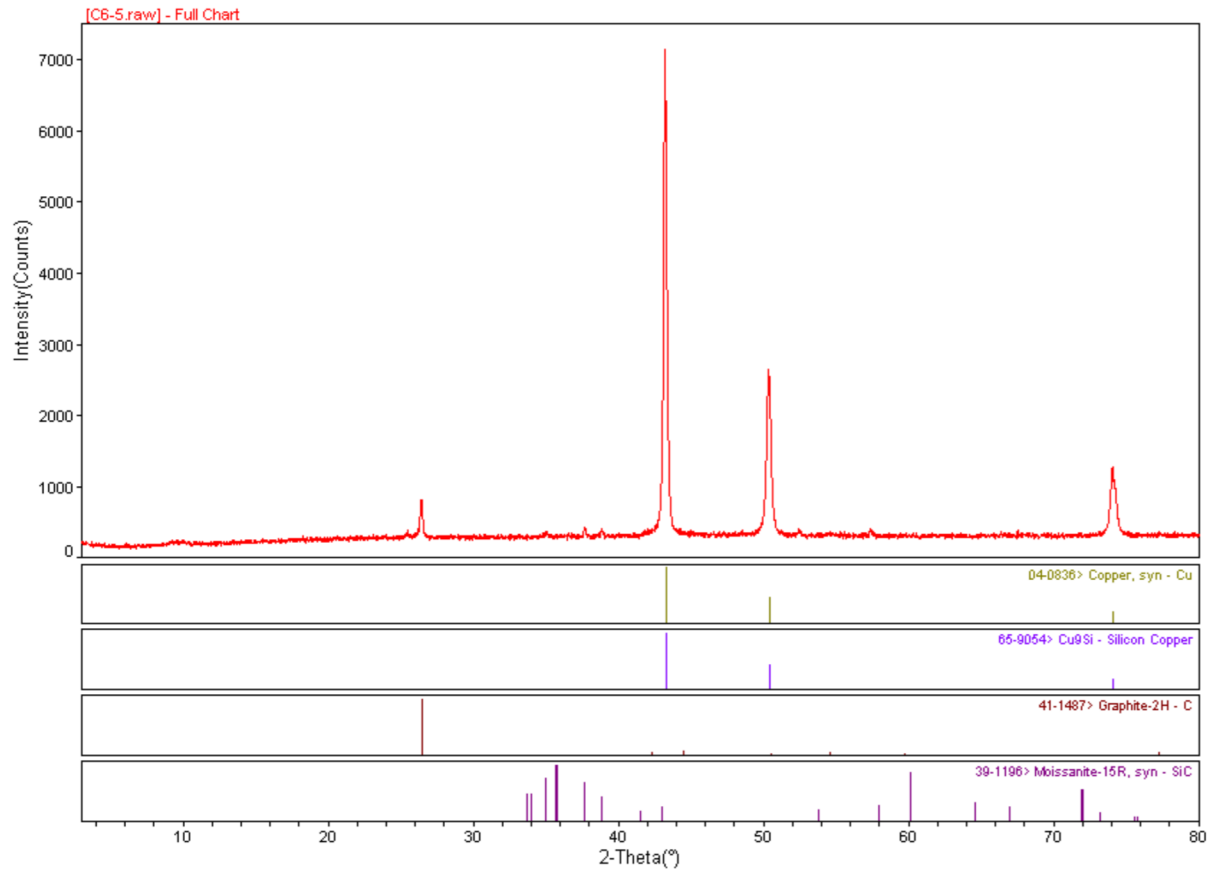

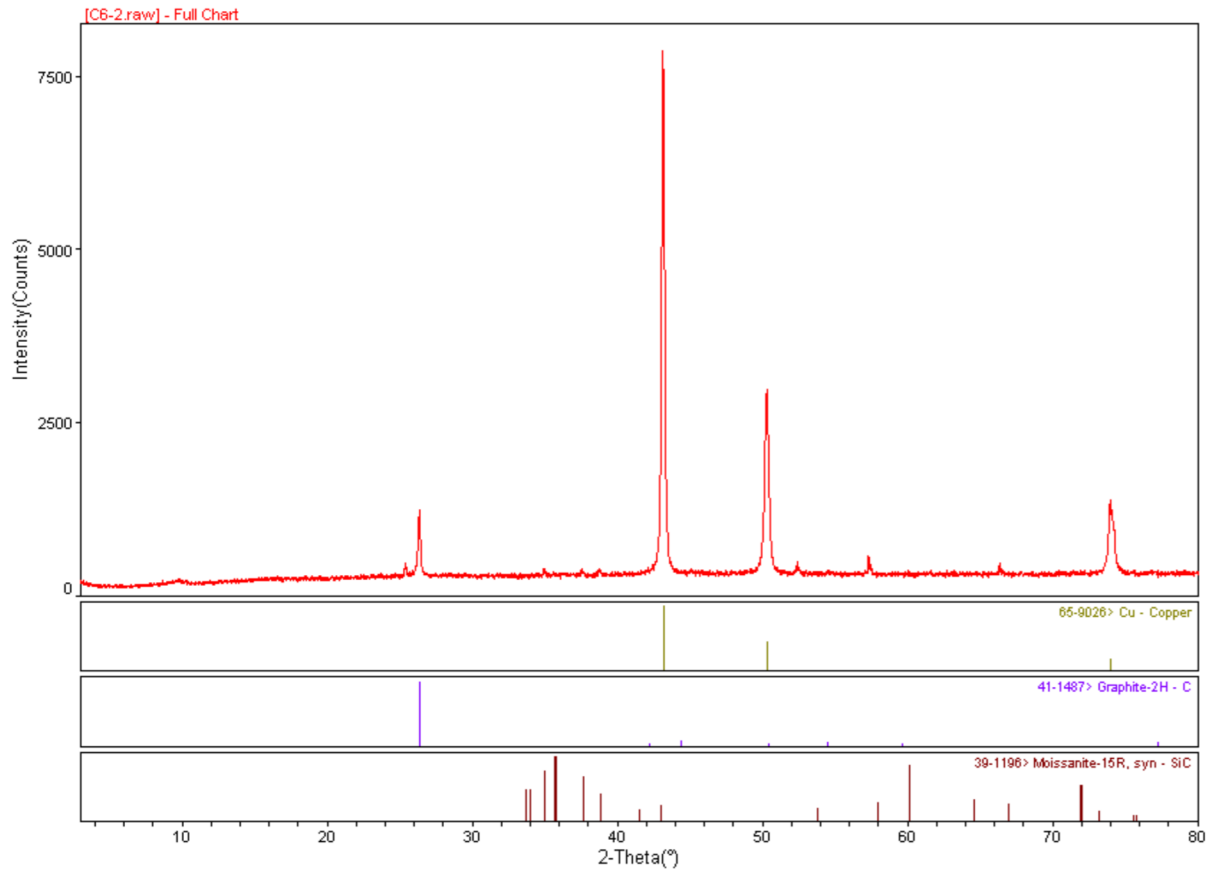

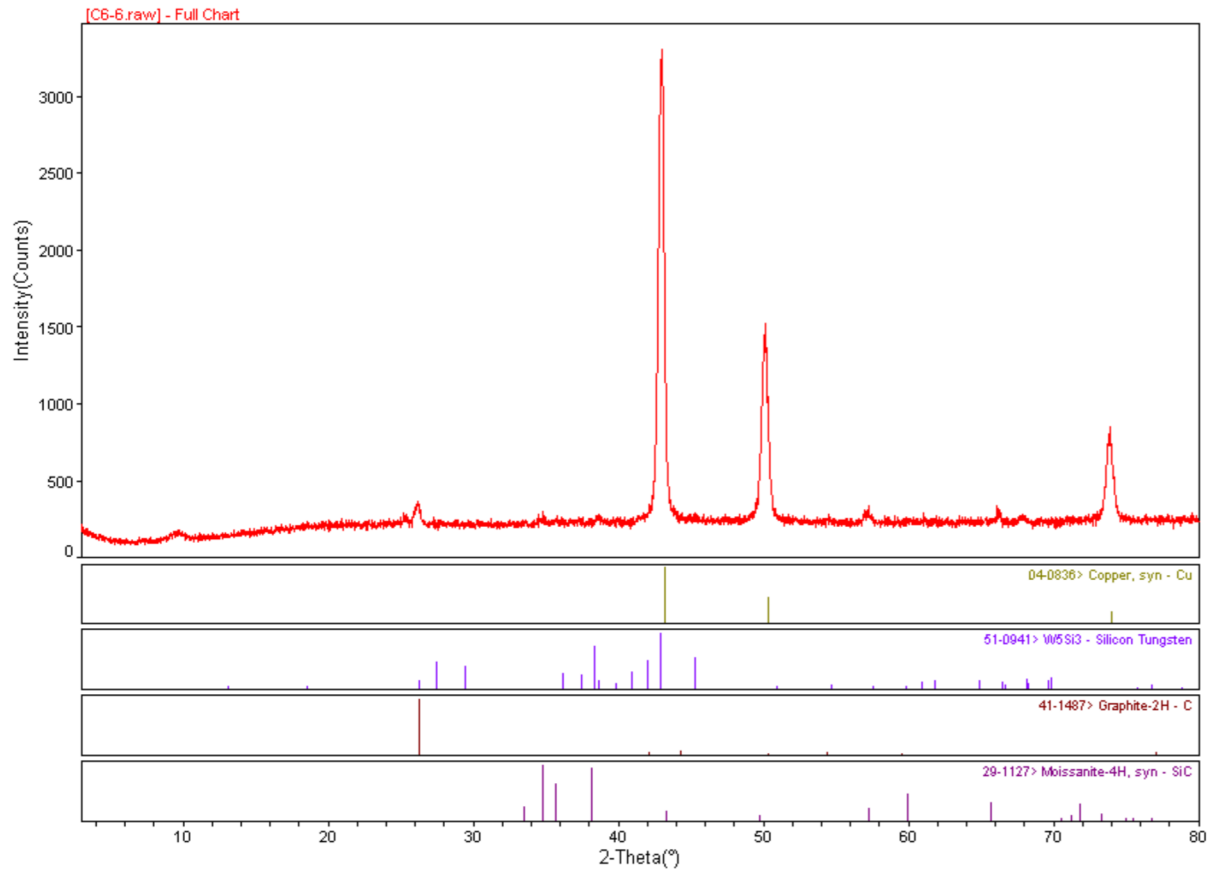

Supplement: Supplementary file 1 [file nanomaterials-12-02154-s001.zip › nanomaterials-1764414-supplementary.pdf]
